# Intact Detection of Highly Occluded Immature Tomatoes on Plants Using Deep Learning Techniques

**DOI:** 10.3390/s20102984

**Published:** 2020-05-25

**Authors:** Yue Mu, Tai-Shen Chen, Seishi Ninomiya, Wei Guo

**Affiliations:** 1 Plant Phenomics Research Center, Jiangsu Collaborative Innovation Center for Modern Crop Production, Nanjing Agricultural University, No. 1 Weigang, Nanjing 210095, China; snino@g.ecc.u-tokyo.ac.jp; 2 International Field Phenomics Research Laboratory, Institute for Sustainable Agro-ecosystem Services, The University of Tokyo, 1-1-1 Midori-cho, Nishi-Tokyo, Tokyo 188-0002, Japan; 3 Graduate School of Agricultural and Life Sciences, The University of Tokyo, 1-1-1 Yayoi, Bunkyo, Tokyo 113-8657, Japan; chen.taishen@ut-biomet.org

**Keywords:** precision horticulture, deep learning, image analysis, robotic harvesting

## Abstract

Automatic detection of intact tomatoes on plants is highly expected for low-cost and optimal management in tomato farming. Mature tomato detection has been wildly studied, while immature tomato detection, especially when occluded with leaves, is difficult to perform using traditional image analysis, which is more important for long-term yield prediction. Therefore, tomato detection that can generalize well in real tomato cultivation scenes and is robust to issues such as fruit occlusion and variable lighting conditions is highly desired. In this study, we build a tomato detection model to automatically detect intact green tomatoes regardless of occlusions or fruit growth stage using deep learning approaches. The tomato detection model used faster region-based convolutional neural network (R-CNN) with Resnet-101 and transfer learned from the Common Objects in Context (COCO) dataset. The detection on test dataset achieved high average precision of 87.83% (intersection over union ≥ 0.5) and showed a high accuracy of tomato counting (R^2^ = 0.87). In addition, all the detected boxes were merged into one image to compile the tomato location map and estimate their size along one row in the greenhouse. By tomato detection, counting, location and size estimation, this method shows great potential for ripeness and yield prediction.

## 1. Introduction

Tomatoes are the second most important horticultural crop [1] in terms of yield, with total production of more than 180 million tonnes across the world (FAO STAT 2017 [2]). The cultivation of tomatoes is one of the most profitable agricultural businesses because tomatoes are self-compatible and have a short life cycle [3]. For high yield and good quality, the crop needs precision management of water throughout the growing period [4], as well as fertilizer and pest control [5]. For example, due to the nonuniform flowering and ripening stages, precise irrigation is needed to ripen immature plants and avoid damage to mature plants [4]. In addition, depending on the ultimate use of the tomatoes, they may be harvested at different stages of ripeness. Tomatoes with “mature green” (medium green to light green) and dim pink colour are shipped, those with reddish-pink colour are sold locally, and those with dark red colour are processed [6]. Thus, optimal tomato cultivation requires tomato-on-plant detection to provide the fruit location and ripening status on spatial variation on which to base agronomic decisions [7]. To inform harvest resourcing and management, and marketing, tomato yield prediction requires dynastically and precise monitoring of tomato number, size, and ripening status [8,9,10]. The combination of computer vision and the Internet of Things (IoT) makes it possible to accurately monitor the growth of greenhouse crops [11]. In addition, as the agricultural population decreases and ages, robots are being considered as replacements for humans to undertake manual and tedious tasks such as harvesting. Harvesting robots perform harvesting actions after collecting and analysing the information from their surroundings [12]. Hence, robot-assisted tomato harvesting also requires the detection of the target fruit. 

Thus, whether for tomato growth monitoring, yield prediction, or for robotic harvesting, tomato fruit detection is a crucial step. In regard to immature on-plant tomato detection in images, there are two main challenges. One is the similarity in colour of an immature tomato to the leaf and vine. For green tomatoes, the aiming pixels cannot be easily segmented using the threshold method or using the calculation of the RGB components [13]. To solve this problem, machine learning was used to mine colour features to help pixel-based classification. By applying a decision tree segmentation model on 15 transformed colour features, immature tomatoes were successfully separated from leaves, stems, and backgrounds [14]. However, it needed blob-based segmentation to further reduce misclassifications due to similar colour. Another method is the use of multispectral sensors to evaluate differences in reflectance to separate fruit from background. For immature green citrus detection, through combining colour and thermal images, an increase in recall from 78.1% to 90.4% and an increase in precision from 86.6% to 95.5% were found [15]. In addition, by using imagery fused with colour (RGB) and near-infrared (NIR), the F1 scores were increased [16]. Nonetheless, a multispectral camera is costly compared with an RGB camera and is less applicable in large-scale farms.

The other challenge for tomato on plant detection is occlusion issue, i.e., tomatoes overlap or are occluded by foliage and vines. X-means clustering algorithm was thus used to detect the position of each tomato in a multi-fruit blob [14]. However, the algorithm depends on the overexposed region of the tomato surface, which is inconspicuous under natural illumination conditions. Some other researchers attempted to detect citrus on trees by valid contour selection and occlusion recovery [17], but this method requires a relatively long fruit contour length, which is not suitable for highly occluded situations. Then sliding window was used to detect individual tomatoes by extracting the Haar-like features in the sub-window and classifying tomato pixels with the AdaBoost classifier [18], but testing needs to be done to determine the optimal sub-window size and sliding step. Therefore, researchers tried building a region of interest pyramid to adapt to different tomato sizes and then detected tomatoes using histograms of oriented gradients and a pretrained support vector machine (SVM) classifier [19]. Nonetheless, for overlapping or occluded areas, the miss rate was as high as 16%.

To overcome these challenges, deep learning techniques are a good choice even when using RGB cameras. Deep learning can perform classification and make predictions particularly well, being flexible and adaptable for a wide variety of highly complex challenges, such as varying illumination and depth, overlapping, and occlusion. Rahnemoonfar and Sheppard [20] presented a simulation-based deep learning method for fruit counting using a modified version of the Inception-ResNet architecture. Chen et al. [21] used another neural network and linear regression to estimate and sum the number of fruits in blobs detected by a fully convolutional network. Other approaches employ the faster region-based convolutional neural network (R-CNN) model not only to count fruits and vegetables but also to locate their position in the image by means of bounding boxes, which is important for automatic harvesting. Moreover, most of the research works that have incorporated popular deep learning architectures took advantage of transfer learning [22], as sometimes it is not possible to train a network from scratch due to a small training dataset or a complex multitask network. Sa et al. [16] deployed DeepFruits using faster R-CNN with VGG-16 through transfer learning. Bargoti and Underwood [23] also used faster R-CNN with VGG-16 and found that compared to transferring weights between orchards, data augmentation yields significant performance gains. However, previous research relating fruit location achieved high accuracy with a relatively low intersection over union (IoU) threshold compared with the common threshold (IoU ≥ 0.5) in object detection, which increased the error in estimation fruit location and fruit size [16,23]. In order to improve the detection accuracy, the deep residual learning was one option, as it solved the “vanish gradient” problem and obtained a 28% relative improvement on the Common Objects in Context (COCO) object detection dataset [24] by reformulating the layers as learning residual functions with reference to the layer inputs. Based on that, some improved deep learning frameworks combined with residual networks appeared, such as Inception-Resnet-v2, which was designed to reap all the benefits of the residual approach while retaining inception architecture’s computational efficiency [25]. Therefore, combining faster R-CNN with deep residual networks, e.g., Resnet 50 [24] and Resnet 101 [24], and Inception-Resnet-v2 [25] may achieve high detection accuracy with less detection error.

Tomato-on-plant detection is expected for precision cultivation and for robotic harvesting. However, immature tomatoes that are highly occluded on plants are difficult to detect and locate using traditional image analysis methods, especially under natural illumination conditions. In this paper, we attempted to trained a faster R-CNN model combined with deep residual learning in real tomato cultivation scenes to (1) accurately detect all the visible tomatoes in photo regardless of fruit occlusion and lighting conditions, (2) count the tomato load, and (3) compile a tomato location map and estimate the tomato size along one row in a greenhouse for further yield prediction and robot harvesting. 

## 2. Materials and Methods

### 2.1. Image Acquisition and Labelling

**Figure 1:**
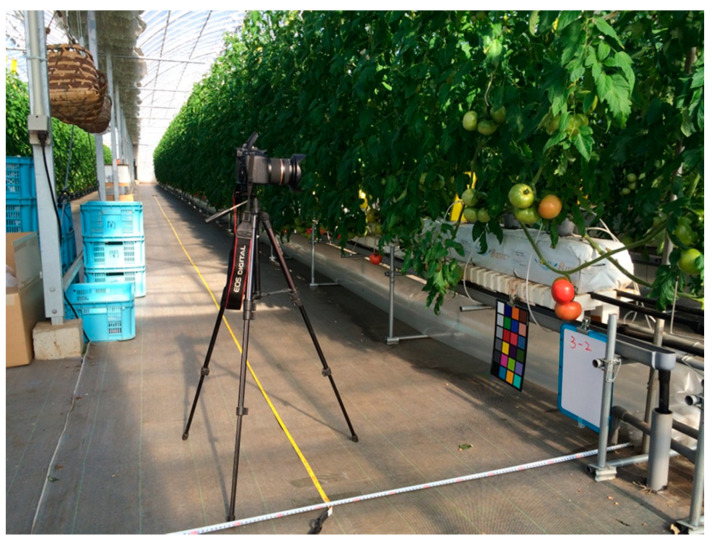
The camera layout for taking tomato photos in the greenhouse of Seki farm.

The photos were collected in two places, the Seki farm and U-Tokyo farm, by a Canon 60D (Canon Inc., Tokyo, Japan) with a Tamron SP10-24 mm lens (Tamron Co., Ltd., Saitama, Japan). The Seki farm is located in Kiyose City, Tokyo, Japan. The photos from the Seki farm were taken on 19 May 2015 and 22 January 2016. There were nine rows of tomato growing on shelves in a greenhouse, and each row had two sides. On each side, photos were taken 0.75 m away from the shelf (Figure 1), and the overlap ratio between two adjacent photos was approximately 0.55. The dimensions of the photos taken on 19 May 2015 were 3456 × 5184 pixels and were vertical in direction, and those on 22 January 2016 were 5184 × 3456 pixels and were horizontal in direction. The Tanashi greenhouse is located at the U-Tokyo farm, Nishi-Tokyo City, Tokyo, Japan. The tomatoes were planted in pots in a greenhouse. The dimensions of all the photos were 5184 × 3456 pixels, and all the photos were taken on 21 January 2016, including 30 photos in the daytime and 32 photos at night. After the photos were collected, they were segmented into three datasets—training, validation, and testing datasets. See details of the dataset in Table 1.

Tomato labelling was implemented through a web-based interactive labelling tool (http://fieldphenomics.com/) developed by the U-Tokyo International Phenomics Research Laboratory. In each photo, all the visible tomatoes were labelled by a bounding box, which were required to be tight enough to cover the object, mainly in the range of 20–70 boxes per image. After that, we checked the image annotation three times by different people. Notably, for the highly occluded tomatoes, the bounding boxes were drawn by the supposed shape depending on the visible part (Figure S1). In total, 640 photos with 28,835 tomatoes were manually labelled. 

### 2.2. Data Pre-Processing

#### 2.2.1. Training and Validation Datasets

The images in the training and validation datasets (Table 2) were used for training the model and accuracy evaluation, respectively. The images were pre-processed by the following four steps: (1) resize the image to 0.5 of its original size; (2) Crop the image into four subimages, considering several factors such as detection accuracy, GPU memory, and convenience of processing; (3) rotate the vertical subimages (864 × 1296 pixels) to horizontal (1296 × 864 pixels) to keep the size of the arrays the same on the two dimensions; and (4) rename the subimages as a digital sequence for processing in TensorFlow.

#### 2.2.2. Test Dataset

**Figure 2:**
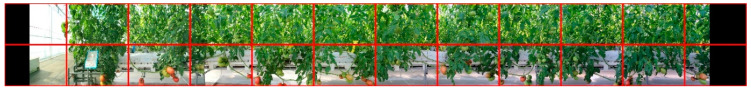
The location of the sub-image in one stitched image of part of the row. Red lines are the outlines of subimages.

One row of the photos was used as test dataset (Table 3) to test the performance of the model in tomato localization. The images were pre-processed using the following five steps: (1) stitch the adjacent photos into six large images using Image Composite Editor (Microsoft Corporation, Redmond, Washington, USA); (2) pad the image on the left and right sides; (3) resize the image to 0.5 of its original size; (4) crop the image by a fixed size of 1296 × 864 pixels, as shown in Figure 2; and (5) rename the subimages as digital sequences for processing in TensorFlow.

### 2.3. Tomato Detection Model Generation

#### 2.3.1. Train Multiple Tomato Detection Models and Select the Model with the Highest Accuracy

We chose faster R-CNN as the tomato detection architecture because of its high precision and speed [26]. Faster R-CNN works as follows: (1) run the image through a CNN to obtain a feature map; (2) run the activation map through a separate network, called the region proposal network (RPN), that outputs boxes/regions; and (3) for the boxes/regions from RPN, use several fully connected layers to output class and bounding box coordinates [26].

The tomato detection model was built on a TensorFlow object detection API (https://github.com/tensorflow/models/tree/master/research/object_detection, Google Inc., Santa Clara, CA, USA) on one workstation with a NVIDIA Tesla P40 graphics card (22919 MB RAM, NVIDIA Corporation, Santa Clara, CA, USA) and Intel® Xeon® CPU E5-2640 v4 (Intel Corporation, Santa Clara, CA, USA), with 503.8 GB RAM, running a 64-bit Ubuntu 16.04 LTS operation system (Canonical Ltd., London, UK).

The faster R-CNN models were pretrained on the COCO dataset with Resnet-50 [24], Resnet-101 [24], and Inception-Resnet-v2 [25] convolutional neural network models, respectively. Because all of these models have shown high accuracy and speed on the COCO dataset, we wanted to investigate their performance on our dataset. With 1779 subimages as training data, 511 subimages as validation data, a learning rate of 0.00003, and random horizontal flip as the data augmentation method, these three models were trained by transfer learning and obtained an average precision (AP) of intersection of union (IoU) ≥ 0.5 in 100 epochs on the validation dataset. Other hyperparameters used the default settings in the faster R-CNN configure file. By comparing the AP, we selected the model with the highest accuracy as tomato detection model.

#### 2.3.2. Detect Tomatoes Using the Selected Model on the Test Dataset

The hyperparameters such as learning rate has been fine tuned to increase the accuracy of the validation data. To test whether the model overfit, the model was applied to the test dataset. The detection was performed by the following five steps: (1) export the TensorFlow graph of the specified checkpoint of the highest AP in the selected model; (2) infer detections from the test dataset with the exported TensorFlow graph with non-maximal suppression threshold of 0.6; (3) evaluate the detections with manually labelled tomatoes as reference and obtain the AP of IoU ≥ 0.5; (4) filter out the boxes with confidence scores below 0.5; and (5) record the detected tomato number and locations.

### 2.4. Evaluation Metrics

The average precision with IoU thresholds of 0.5 was used to quantify the model accuracy. This metric is popular for measuring the accuracy of object detectors, as it balances the performances of precision and recall. The average precision could be calculated by the area under the precision-recall curve. Precision is calculated by Equation (1), and recall is calculated by Equation (2), where *TP* is the number of true positives, *FP* is the number of false positives, and *FN* is the number of false negatives. Both of these parameters are calculated on all the boxes by rank according to the descending predicted confidence.

(1) $$Precision=\frac{TP}{TP+FP}$$

(2) $$Recall=\frac{TP}{TP+FN}$$

In this research, we used COCO detection metrics to calculate the average precision. The COCO metrics are the official detection metrics used to score the COCO competition (http://cocodataset.org/) and can report statistics such as AP at IoU thresholds of 0.5.

### 2.5. Tomato Localization

Then, tomatoes detected in subimages was composed together and located in the stitched image. The tomato localization was performed by the following five steps: (1) rename the detected sub-image to its original name for linking to the original image; (2) put the detected sub-image to the original location of the stitched image; (3) recalculate the box coordinates in the stitched image and label the boxes on the edge of the subimage; (4) Merge the boxes on the seams by recognizing the split parts using the box distance and shape similarity (ratio of the side length in both directions); and (5) calculate the number of tomatoes in each stitched image and outline the detected boxes.

### 2.6. Tomato Size Estimation

Finally, tomato sizes in images including width, height, and aspect ratio were determined by the bounding box merged in the location map. The tomato width and height were represented by the bounding box width and height, and the aspect ratio was calculated by the ratio of the width to the height. 

## 3. Results

### 3.1. Accuracy Analysis of Deep Learning Models

**Figure 3:**
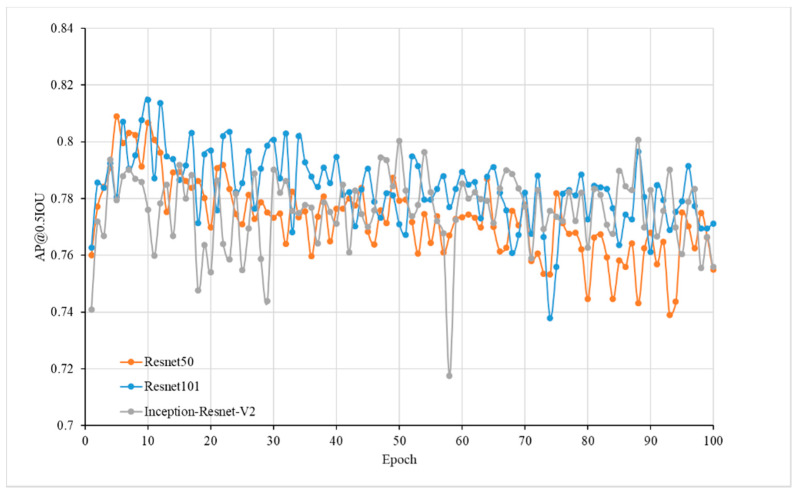
Accuracy of the tomato detection model.

There were three deep learning models trained with different deep convolutional neutral networks, i.e., Resnet 50, Resnet 101, and Inception-Resnet-v2. Running the models on the validation images yielded the change in average precision with the epoch (Figure 3). It was observed that all the models obtained the highest accuracy at approximately 10 epochs, and then the accuracy decreased. This may be caused by overfitting, as the size of training dataset was quite small compared with the COCO dataset. Among the models, the deep learning model with Resnet 101 achieved the highest AP of 0.82, so this model was selected as the tomato detection model.

Then, the model was applied on the test dataset and achieved the AP of 0.87. Then, the detected results were further analysed with the distribution of true positive, false positive, and false negative at an IoU threshold of 0.5. True positive means that a box was detected as a tomato and overlapped with one manually labelled tomato box, and the interception area was greater than 0.5 of the union of their areas. False positive means a box was detected as a tomato, but its IoU was less than 0.5 with any manually labelled tomato box. False negative means a box was not detected as a tomato, but its IoU was greater than 0.5 with one or more manually labelled tomato boxes. Therefore, the false positive number is the number of detected tomatoes that are not tomatoes or that do not meet the IoU requirements, and the false negative number is the number of missed tomatoes or those that were not accurately located. 

**Figure 4:**
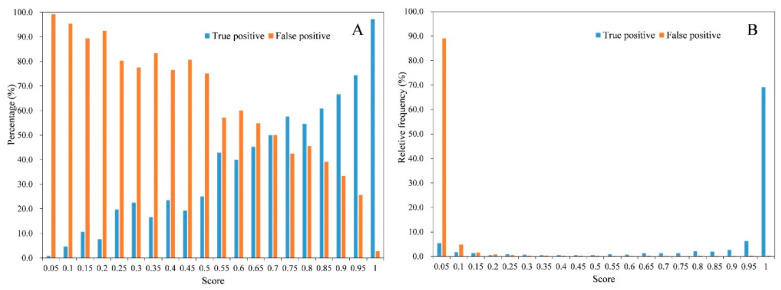
Distribution of true positives and false positives with scores by applying the tomato detection model on the test dataset. (**A**) Percentage change of true positives and false positives with scores; (**B**) relative frequency change of true positives and false positives.

**Figure 5:**
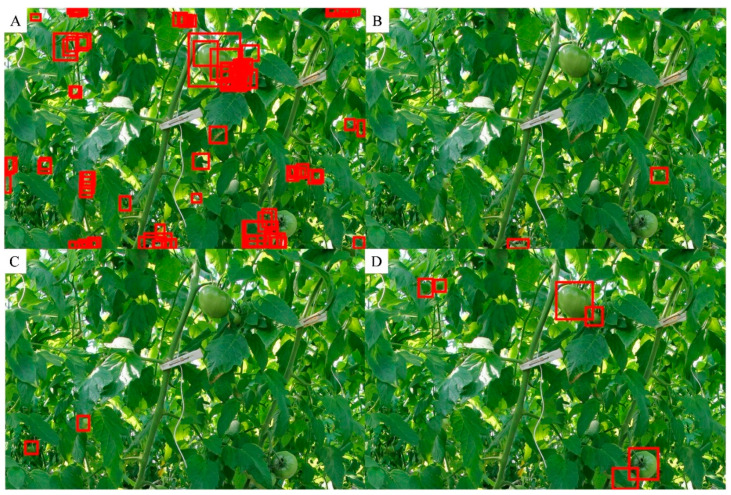
Examples of detected tomatoes with different scores in the test dataset. Subfigure (**A**) shows boxes with scores ≤ 0.3, (**B**) shows boxes with 0.3 < scores ≤ 0.5, (**C**) shows boxes with 0.5 < scores < 0.7, and (**D**) shows boxes with scores > 0.7.

We counted the number of true positives and false positives based on the detected tomato box and its scores. Figure 4A shows that as the score increased, the percentage of false positives decreased. At the same time (Figure 4A), the percentage of true positives increased, which means that more detected tomato boxes met the requirement of IoU. The relative frequency distribution (Figure 4B) showed that more than 80% of the false positives had scores less than 0.1, and more than 80% of the true positives had scores not less than 0.9. As shown in Figure 5A, when the score was low, there were many overlapping boxes with poor location precision. This may lead to many false positive detections at IoU ≥ 0.5. Comparing the tomato sizes in Figure 5C and Figure 5D indicated that large size tomatoes tended to have high scores. However, there was a small portion of manually labelled tomatoes with scores less than 0.2.

### 3.2. Tomato Counting Assessment

**Figure 6:**
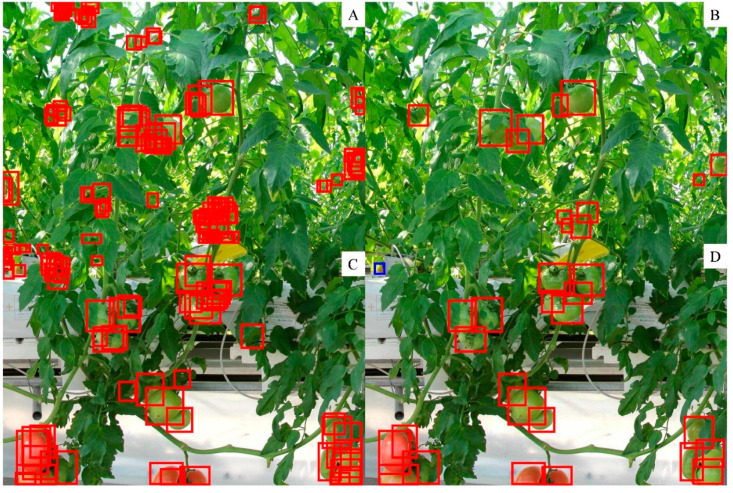
Example of all detected tomatoes (**A**,**C**) and filtered detected tomatoes with a score ≥ 0.5 in the test dataset (**B**,**D**).

**Figure 7:**
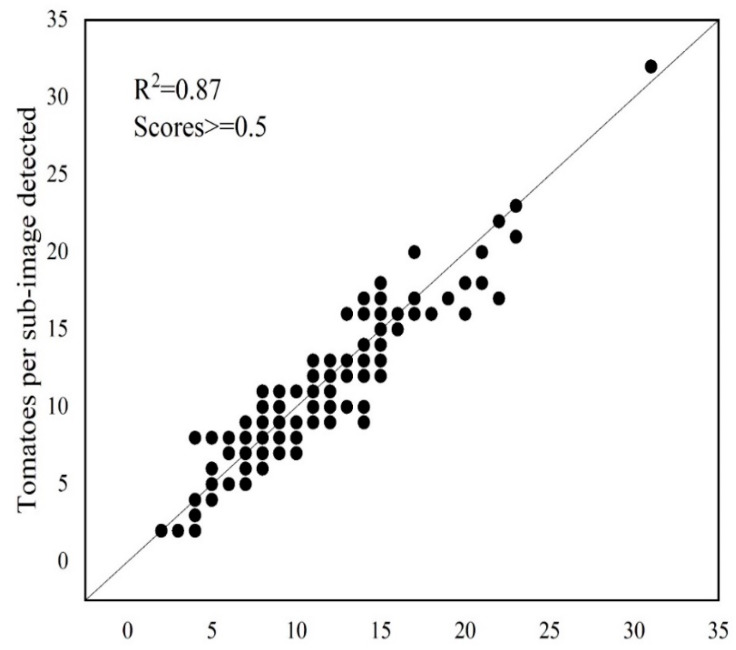
Correlation between labelled and detected tomatoes per subimage in the test dataset.

Tomato counting is the basic step for yield estimation [9,21]. Through the application of the selected deep learning model to the test dataset, tomatoes were detected, and the boxes of tomatoes in each subimage were labelled (Figure 6A,C). According to the distribution of true positives (Figure 4), the detected tomatoes were filtered by score. Then, only the tomatoes with scores ≥ 0.5 were retained (Figure 6B,D), and the number of tomatoes in each subimage was recorded. Correlation analysis of the number of labelled and detected tomatoes per subimage showed a high coefficient of determination (R^2^ = 0.87). However, the number was slightly underestimated when the tomato number was greater than 20 per subimage, as shown in Figure 7. This may be caused by some tomatoes with low scores (less than 0.5), which were filtered out. One example is the tomato in the image shown in the blue box of Figure 6D, of which less than a quarter was visible and was quite small, but it has been manually labelled.

### 3.3. Tomato Localization for the Whole Cultivation Bed

**Figure 8:**
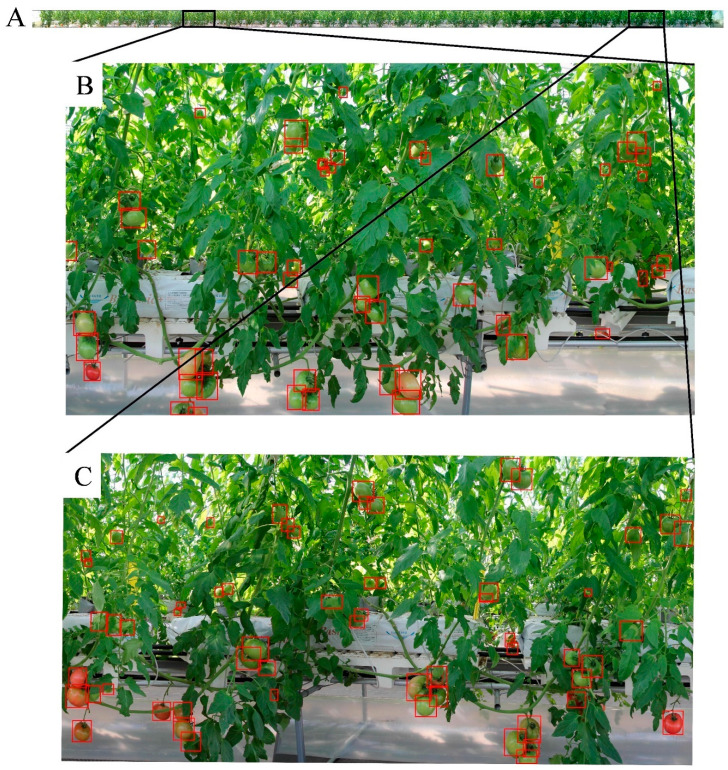
Detected bounding boxes of the tomatoes in the stitched image. (**A**) is the location map for tomatoes in one row; (**B**,**C**) are two enlarged zones in (**A**) for clear illustration.

Yield mapping is quite important for monitoring the growth of tomatoes, providing information on spatial variation on which to base agronomic decisions [7]. Precise tomato detection allows for the generation of yield maps and could also be an assistant tool for robotic harvesting. After the tomatoes in the subimages were detected and labelled, the tomatoes were transferred to the stitched image of one cultivation row in the green house, and the segmented box on the seaming line was merged. In total, 1422 tomatoes were detected and located along this row. Figure 8A shows the location map for tomatoes in one row in the greenhouse of the Seki farm. By enlarging some zones in Figure 8A, it shows that the red tomatoes were all near the bottom of the shelf, and the green tomatoes were mainly in the upper region (Figure 8B,C).

### 3.4. Tomato Size Estimation

**Figure 9:**
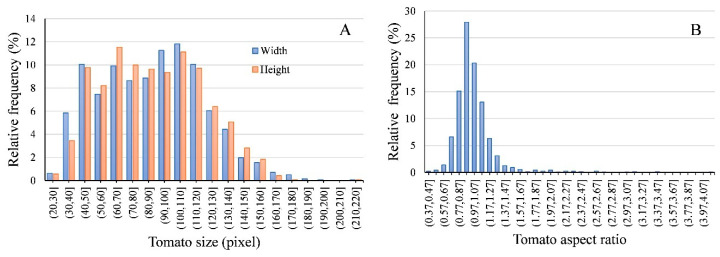
Relative frequency change of the tomatoes size (**A**) and aspect ratio (**B**) in images. Tomato size is represented by box width and box height. Tomato aspect ratio is calculated by width/height.

Tomato size estimation is of great significance for yield estimation [7,27] and prediction [8,10]. On-tree estimation of fruit size is useful for the prediction of maturity and harvest time [8,10], and estimation of fruit size together with fruit number allows estimation of fruit weight (‘yield’) [7,27]. By using the faster R-CNN models with Resnet-101, the final detection of tomato showed precise detection. Tomato length, width, and aspect ratio in an image can be determined based on the bounding box. Taking the tomato merged in one row (see details in Section 2.5) as an example, the size distribution (Figure 9A) showed that about 16.53% and 13.78% of the tomatoes had a width and height less than 50 pixels, respectively. The aspect ratio (Figure 9B) showed that about 89% tomatoes had an aspect ratio in the range of 0.67 to 1.27, and about 33% tomatoes had an aspect ratio in the range of 0.97 to 1.17. The closer the aspect ratio value was to 1, the closer the shape of tomato was to a circle. 

## 4. Discussion

### 4.1. Accuracy Comparison with Other Tomato-on-Plant Detection Techniques Using RGB Images

Using the faster R-CNN models with Resnet-101, the tomato detection model showed relatively high precision on the test dataset. To compare with previous tomato detection performance, the method and the best result of each paper are listed in Table 4. Following the suggestion by Koirala [7], the F1 score of the test detection in this research was calculated for comparison (see Figure S2). When available, the F1 score of other researches were recorded. Some previous researches lack the F1 score recording or precision-recall curve for determining the F1 score [13,14,18,28], but from their accuracy descriptions, the accuracy of this research were higher than most machine learning method. The research [19] got a high F1 score for mature tomato detection, which was less difficult than for green tomatoes. However, when compared with other deep learning approaches [29,30], this method showed lower accuracy. On one hand, this was due to the lower resolution or smaller size of tomatoes in our image, as we aimed to compile a tomato layout map. On the other hand, both of their studies optimised the non-maximum suppression with IoU, which inspired us for future work.

### 4.2. Error Analysis

#### 4.2.1. Overfitting

**Figure 10:**
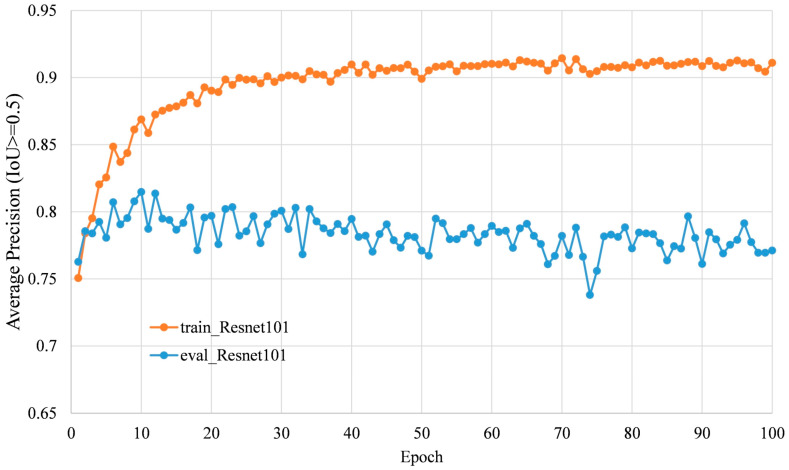
Change in average precision (AP) at an intersection of union (IoU) threshold of 0.5 of training data and validation data using Resnet 101 with the epoch.

Overfitting is a common problem in machine learning. According to observations of the AP with the epoch (Figure 10), the faster R-CNN model with Resnet 101 showed overfitting after epoch over 10. One reason may be the size of training dataset was quite small which consisted of 1779 subimages (864 × 1296 pixels), as the model was pretrained on the COCO dataset, which consisted of 118,287 images and 80 classes for training (https://www.tensorflow.org/datasets/catalog/coco). Enlarging the training data size and diversity may help to solve this problem. Data augmentation techniques [31] could artificially enlarge both the number and the variety of training images and have been shown to yield significant performance gains [23]. In this research, only random horizontal flip was used as the data augmentation method, and more data argumentation methods will be used to get a better training model in the future.

#### 4.2.2. Manual Labelling

Manual labelling served as ground truth in the accuracy analysis. For precise fruit detection, ground truth boxes should be tight enough to cover the object and some of the background around the object perimeter [32]. However, we cannot ensure that all the visible tomatoes were labelled and all the placement for boxes were appropriate, even though we checked the images three times. For the same tomato, different people will label the tomato with a box of different sizes and locations. This causes errors in tomato training and detection. Several efficient labelling methods have been developed [33,34,35]. Use of these methods not only saves time spent labelling but also uses less labour, making it possible to standardize the method of box placement. 

#### 4.2.3. Influence of Tomato Size on Determination

**Figure 11:**
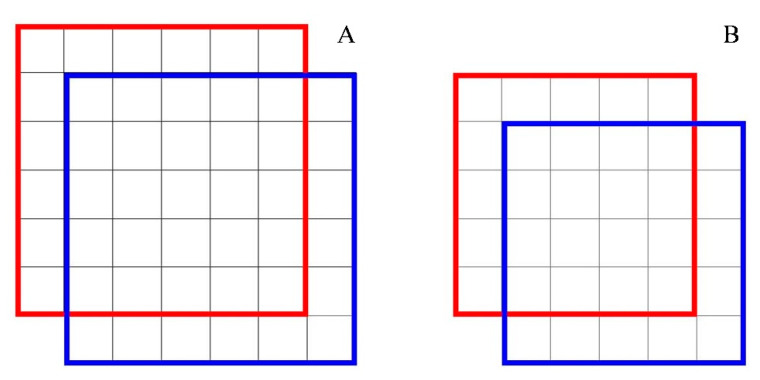
Illustration of the IoU value difference for the same offset in pixels but for different size objects. The IoU of the left boxes (**A**) is 0.53, and the IoU of right the boxes (**B**) is 0.47. Although both of them has the same offset (one pixel) in the same direction, the left detection is true positive while the right one is false positive with IoU threshold of 0.5.

**Figure 12:**
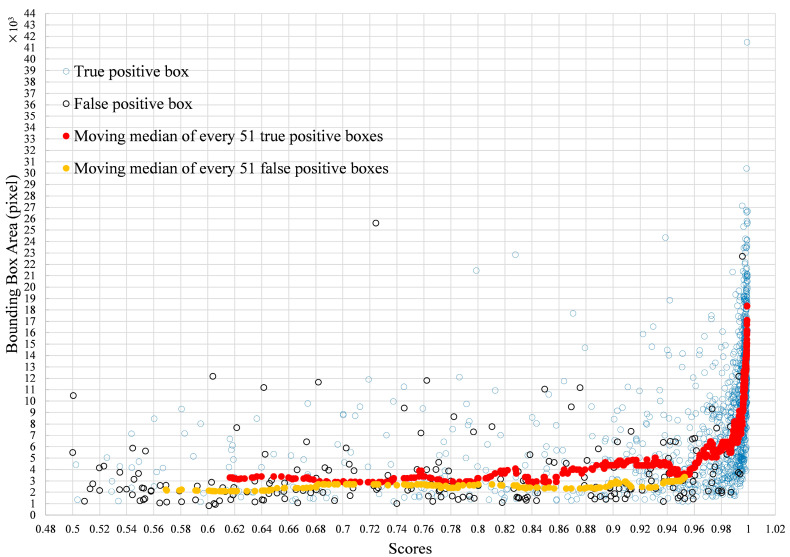
Area distribution of detected true positive boxes and false positive boxes with the scores, and their moving median value with the scores.

The results (Figure 6D) showed that very small tomatoes (in image size) may have a higher possibility regarding false or missing detection. Other than less information increasing the difficulty in recognition, it was partly due to the criterion for determining truth positive. The detection was recognized as true positive only if it has enough overlap with the manually label box measured by IoU. Based on the IoU calculation equation, the smaller the object is, the lower the IoU value when offset in pixels. As shown in Figure 11, the IoU of the left boxes (Figure 11A) is 0.53, and the IoU of the right boxes (Figure 11B) is 0.47. This means that for very small tomatoes, a small misplacement of the box causes a low IoU. Hence, even small errors in the detection of smaller fruit caused these fruits to be registered as false positives [23]. As Figure 12 showed, by calculating the moving median value of every 51 true positive boxes and false positive boxes, respectively, the box area of true positive was a little larger than false positive at all the scores. The box area of 28.99% of the false positive boxes was smaller than 2000 pixels, and 81.16% of the false positive boxes was smaller than 5000 pixels, while that of only 5.59% and 28.33% of the true positive, respectively (Table 5). Therefore, the detector seems to work better for tomatoes with a bounding box area large than 2000 pixels and could largely reduce false positives for tomatoes with a bounding box area large than 5000 pixels. This issue could be solved by increasing the image resolution in the future.

### 4.3. Limitations

#### 4.3.1. Long Training Time

We used transfer learning for tomato detection, which has some shortcomings in training time compared with other machine learning methods, e.g., support vector machine and random forest methods. However, compared with the training time, the testing time cost of detection per image is more important for application. We tested the model on one mini-size ZBOX computer (ZOTAC, Shenzhen, China) that was equipped with an Intel® Core™ i5-7500T CPU (Intel Corporation, Santa Clara, CA, USA), NVIDIA GeForce GTX 1060 (NVIDIA Corporation, Santa Clara, CA, USA) and 64-bit Ubuntu 16.04 operation system (Canonical Ltd., London, UK). The model required 0.37 s to detect tomatoes per image, which is acceptable for real-time detection. In addition, if we take into account the time needed to manually design filters and extract features, “the time used for annotating images and training the CNN becomes almost negligible” [36].

#### 4.3.2. Only Visible Tomatoes

A fruit load estimation relies on the assessment of the total number of fruits per tree, not the number of fruit visible in an image [32]. As this method is based on RGB images, it could only detect the tomatoes visible in the image. If the tomatoes are entirely shaded by leaves or other tomatoes, then they cannot be counted. Actually, the tomato growing “wall” is 3D, but we can only see two dimensions from the image. In the future, by increasing the number of image viewpoints by taking photos on both sides of the row and merging the tomatoes detected from both sides of the row, we could reduce the influence of the nonvisible and get the real tomato counts. 

#### 4.3.3. Tomato Size Estimation in Images

The tomato size measured in real-world dimensions such as in centimetres is more important for estimating tomato yield. However, in this research, we only used one camera and obtained 2D images, while the tomatoes in the same image has different distances to the camera considering that the tomato was not located on the same plane and the effects of camera projection. This means that we should not use a fix conversion parameter from image size to real size. In the future, we will consider using a multi-vision camera to generate depth information to get the distance of each object to the camera, and this would assist in estimating tomato real size as a conversion reference from tomato image size.

### 4.4. Perspectives on Ripeness Estimation and Yield Prediction

**Figure 13:**
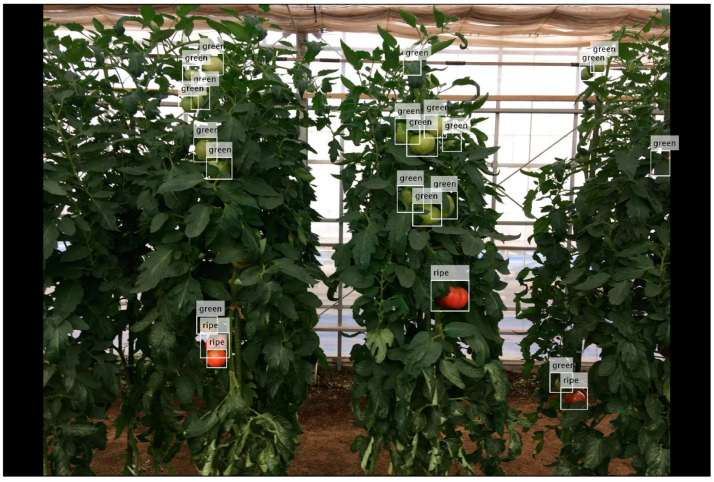
Application of tomato detection in ripeness estimation. The tags labelled ripe or immature tomatoes.

As each tomato was labelled by a bounding box, the ripeness of the tomatoes could be estimated. The tomatoes could be cropped by a bounding box and compared with reference tomato images. Then, by using customized bag of colour layout features, their similarity could be assessed. All the detected tomatoes had a similarity score in the range of zero to one, with more similarity to the referential tomato for a higher score. There is an example shown in Figure 13.

Deep learning has yet to be applied to for prediction of fruit load per tree and per orchard [32]. However, in crop yield prediction such as corn [37] or soybean [38], it already has some attempts. By combining time series of tomato growth data (i.e., tomato size, number and ripeness status) and the environmental data (e.g., weather, soil data) collected in internet of things enabled greenhouse [11], as well as diseases detection [39], using deep learning to build a long short-term memory model with convolution neutral network has great potential in yield prediction.

## 5. Conclusions

In this paper, we trained three faster R-CNN models combined with three deep residual networks (i.e., Resnet 50, Resnet 101, and Inception-Resnet-v2) for tomato detection. The tomato detection model using faster R-CNN with Resnet 101 achieved the highest average precision and was selected for tomato detection. It got average precision of 87.83% (IoU ≥ 0.5) on test dataset, showing good accuracy for detecting highly occluded immature tomatoes on plants in real cultivation scenes. For tomato counting, it received a high coefficient of determination (R^2^ = 0.87) with tomato manually labelled considering more than 10% quite small tomatoes (size < 50 pixels). By locating the tomatoes and merging the split tomatoes, 1422 tomatoes were detected and located along one tomato cultivation row in the greenhouse, and their sizes were estimated based on the bounding box. By tomato detection, counting, location, and size estimation, this method shows great potential for ripeness and yield prediction.

## Figures and Tables

**Table 1:** Details of the dataset.

Location	Date	Time	Photo Number	Photo Size (pixels)
Seki farm	19 May 2015	Day	229	3456 × 5184
	22 January 2016		349	5184 × 3456
Tanashi green house	21 January 2016	Day	30	
		Night	32	

**Table 2:** Details of the training and evaluation datasets.

Dataset	Location	Date	Time	Photo Number	Subimage Number	Image Size (pixels)
Train dataset	Seki farm	19 May 2015	Day	452	1779	864 × 1296
		22 January 2016				1296 × 864
	Tanashi green house	21 January 2016	Day			
			Night			
Evaluation dataset	Seki farm	22 January 2016	Day	129	511	
	Tanashi green house	21 January 2016	Day			
			Night			

**Table 3:** Details of the test dataset.

Dataset	Location	Date	Time	Photo Number	Subimage Number	Image Nize (pixels)
Test dataset	Seki farm	22 January 2016	Day	59	135	1296 × 864

**Table 4:** Scientific reports in tomato on plant detection based on colour (RGB) images. The best result of each paper is shown. When available, the F1 score is recorded; otherwise, the validation metric used by the authors is included.

Author	Method	Accuracy
Schillaci et al., 2012 [28]	Scanning window with support vector machine	Twenty true positives against 26 false positive
Khoshroo et al., 2014 [13]	Colour analysis and region growing	Overall classification accuracy: 82.38%
Yamamoto et al., 2014 [14]	Pixel-based segmentation, blob-based segmentation, X-means clustering	Recall: 0.8, precision: 0.88
Zhao et al., 2016 [18]	AdaBoost classifier and colour analysis	True positives rate: 96.5% False positive rate: 10.8% Missing (False negative) rate: 3.5%
Sun et al., 2018 [29]	Faster R-CNN with Resnet 50	mAP (green and red tomatoes): 90.9%
Liu et al., 2019 [19]	Machine learning and colour analysis	F1 score: 92.15%
Liu et al., 2020 [30]	Yolo-tomato	F1 score: 93.91%, AP: 96.40%
This paper	Faster R-CNN with Resnet 101	F1 score: 83.67%, AP: 87.83%

**Table 5:** Cumulative relative frequency of true positive and false positive boxes of box area.

Box Area (Pixel)	True Positive Boxes	False Positive Boxes
≤1000	0.00%	0.97%
≤2000	5.59%	28.99%
≤3000	13.97%	59.42%
≤4000	20.83%	72.95%
≤5000	28.33%	81.16%

## Supplementary Materials

The following are available online at https://www.mdpi.com/1424-8220/20/10/2984/s1, Figure S1. Example of tomato bounding box labelling by a web-based interactive labelling tool (http://fieldphenomics.com/); Figure S2. Precision-recall curve of the tomato detection model on test dataset.
