# Supplementary material for: Intact Detection of Highly Occluded Immature Tomatoes on Plants Using Deep Learning Techniques
Source: Sensors (Basel). 2020 May 25;20(10):2984. doi: 10.3390/s20102984 (PMC7288109; doi:10.3390/s20102984)
Supplement: Supplementary file 1 [file sensors-20-02984-s001.pdf]

Sensors **Supplementary File**

Article title: **Intact detection of highly occluded immature tomatoes on plants using deep learning techniques**

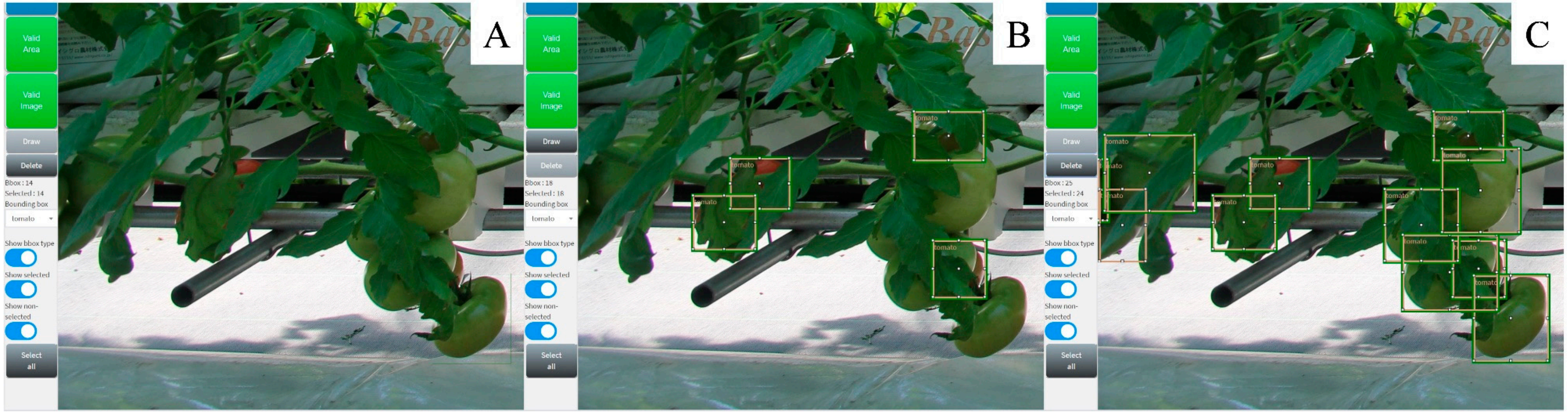

Figure S1. Example of tomato bounding box labeling by a web-based interactive labelling tool (http://fieldphenomics.com/). Subfigure A shows the image before annotation, B shows the labeling of highly occluded tomatoes which was drawn by the supposed shape depending on the visible part, C shows the final labeling of all tomatoes.

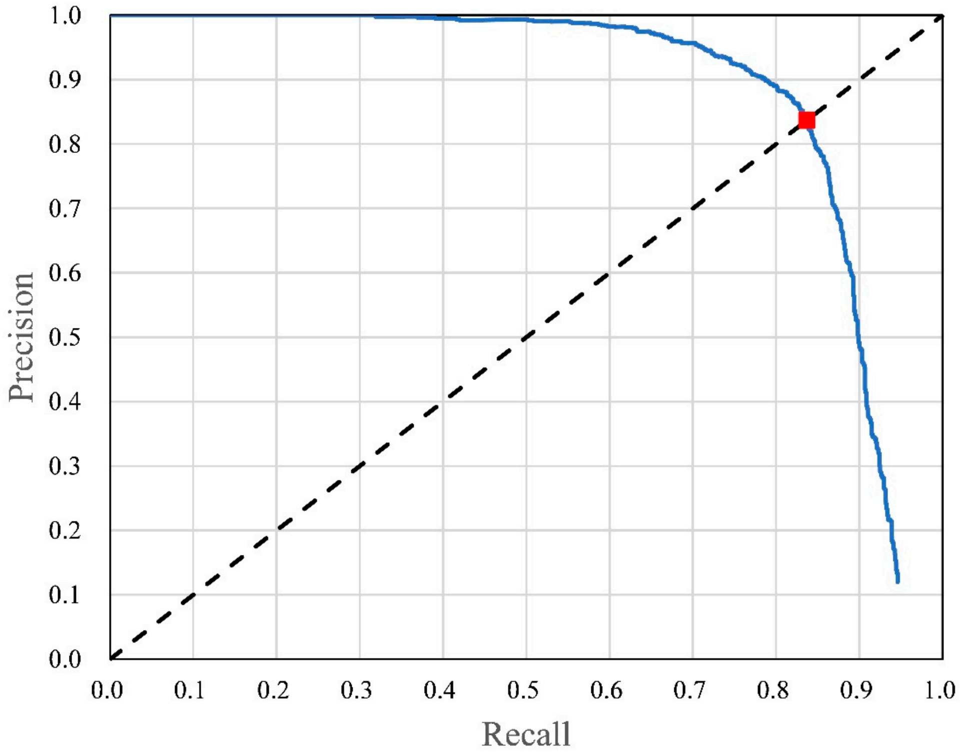

Figure S2. Precision-recall curve of the tomato detection model on test dataset. The marks indicate the point where precision and recall are identical, and F1 scores are computed at this point.
